# Supplementary material for: COVID-19 Vaccine Hesitancy among English-Speaking Pregnant Women Living in Rural Western United States
Source: Vaccines (Basel). 2023 Jun 16;11(6):1108. doi: 10.3390/vaccines11061108 (PMC10304157; doi:10.3390/vaccines11061108)
Supplement: Supplementary file 1 [file vaccines-11-01108-s001.zip › vaccines-2412965-SI.pdf]

Supplementary Material

Table of Contents

Table S1. Direct Interview Guide. 2

Table S2. Thematic Code Book 3

Table S3. Key themes, sub-themes, and quotes from direct interviews. 4

Table S1. Direct Interview Guide.

<u>Pregnancy</u><br>1. How are you feeling about this pregnancy? [Or if post-partum: How was your pregnancy experience?]<br>2. Is this your first pregnancy?<br>3. Where do you find support during your pregnancy [or post-pregnancy]?
<u>Sources of Information</u><br>1. Where do you turn to for information about health decisions during your pregnancy?<br>2. Have any of these sources brought up the COVID-19 vaccine?<br>3. What is your relationship with your primary care provider like? (i.e., do they help you make decisions, do you contact them often, do you trust them, would you go to them for other health care needs or future pregnancies)<br>4. What is their role in helping you make health decisions?<br>5. What do they or can they do to build your trust in them?<br>6. Who are trusted sources of information on the vaccine in your community?<br>7. What have you heard about the COVID-19 vaccine from your community?<br>8. If faith is brought up: What is your relationship with this community like? What do leaders in this community do to earn your trust? To what level do you consider yourself to be religious?<br>9. Who are trusted sources of information on the vaccine in your personal life?<br>10. What influence does this person have on decision making during pregnancy, etc. (for ex, foods, birthing plan, medical decisions)<br>11. What are sources of information that you distrust?<br>12. What is it about these sources that you distrust?
<u>Social media</u><br>1. Do you use social media as a source of information for COVID-19 related information?<br>2. Which sites/ apps do you use?<br>3. What do you like about sources of information on these sites?
<u>Vaccinations</u><br>1. How do you perceive your own risk to COVID-19? [How worried are you about getting the COVID-19 virus?]<br>2. Before, during and after pregnancy?<br>3. Have you received the COVID-19 vaccine?<br>4. If so, what factors influenced your decision to get the vaccine?<br>5. If not, what factors influenced your decision not to get the vaccine?<br>6. What has your experience been with other vaccines? (For yourself/for children?)<br>7. What has prompted you to get specific vaccines? What has prompted you to not get specific vaccines?<br>8. Have you received vaccinations during your pregnancy?<br>9. Has your perspective on vaccines changed in the last two years? (prior to COVID-19 vs post)<br>10. Has this influenced the decisions you've made regarding your children's vaccinations,<br>11. Or how do you talk with your family about vaccinations?<br>12. If not vaccinated: What are reasons for not receiving vaccines for yourself? Do your family members feel the same way?<br>13. Are there any more experiences/stories you would like to share?<br>14. If anything comes up even after this interview, please feel free to reach out to us.

Table S2. Thematic Code Book

Parent Code	Child Code	Description
Pregnancy experience	Positive	Participant refers to a positive pregnancy experience
	Negative	Participant refers to a negative pregnancy experience
	Neutral	Participant feels neutral about their pregnancy experience
Support during pregnancy	Family	Participant refers to family, excluding spouse, being supportive during pregnancy
	Spouse	Participant refers to their spouse as a source of support during pregnancy
	HCP	Participant refers to HCP as a source of support during pregnancy
	Community	Participant references to their community as a source of support during pregnancy
	Friends	Participants refers to friends as a source of support during pregnancy
Relationship with HCP/PCP	Positive	Participant refers to positive experiences with their HCP/PCP
	Negative	Participant refers to negative experiences with their HCP/PCP
Facilitators to Vaccine Uptake		Participant explains facilitators to vaccine uptake, or reasons for getting vaccinated
Barriers to Vaccine uptake		Participant explains barriers to vaccine uptake, reasons they didn't get vaccinated, or reasons they didn't want to get vaccinated even if they ultimately did
Influences on health decision-making	HCP/PCP	HCP/PCP are one of the primary influences on a respondent's health decision making
	Family	Family play an important role in informing health decisions
	Spouse	Spouse plays an important role in informing health decisions
	Media	Media plays an important role in informing health decisions
	Online searches	Online web searches play an important role in informing health decisions
Sources of Information	Trusted sources	Participants referenced their own trusted sources of information on the vaccine or COVID-19
	Distrusted sources	Participants referenced their own distrusted sources of information on the vaccine or COVID-19
Vaccine Misinformation		Participants mentioned hearing vaccine misinformation
Perceived risk of COVID	Self-perceived risk	Participant mentions self-perceived risk of COVID-19
	Perceived risk by others	Participant mentions their perceived risk of COVID-19 from others
Vaccine Hesitancy	Vaccine hesitant	Participant expresses vaccine hesitancy
	Vaccine positive	Participant expressed confidence in vaccines
Ads - Positive feelings		Participants expresses positive feelings toward ads
Ads - Negative feelings		Participant expresses negative feelings toward ads
Ads - Neutral feelings		Participant expresses neutral feelings toward ads

Abbreviations: COVID-19, coronavirus disease 2019; HCP/PCP, health care provider/primary care provider.

Table S3. Key themes, sub-themes, and quotes from direct interviews.

<u>**Vaccine Uptake**</u>

Facilitators

Recommendations from care providers

- "My doctor wasn't...pushy about anything. But when I had questions about it like you know, 'would you recommend this? When would you recommend I get it?' She, you know, gave me that information and, you know, encouraged me to get it."
- "[My decision to get vaccinated] goes back to that one on one conversation with our family doctor, and the research that they found with the studying of the placenta...Had we not had that conversation, and she didn't send me that information...I probably would not have [gotten vaccinated]."

Perceived efficacy

- "I was very convinced by what I had seen regarding its effectiveness and preventing serious illness."
- "[I wanted] to protect [my baby] and myself, and the rest of my family."
- "I'm around elderly patients a lot…I wanted to [get vaccinated] for them and selfishly for me also. I just knew that I would rather have the side effects of the vaccine than actually getting Covid."
- "I had a very young daughter at the time...she was an infant, so I wanted to do whatever I could to protect her from getting it."

Requirements

- "I mostly got the COVID vaccine because…I didn't want to have to deal with...the whole quarantine and stuff…otherwise I wasn't really, necessarily for or against it."
- "I was going to Mexico for my honeymoon. And my husband was like 'You better get vaccinated, so you can come back home, or so that you can actually enter'."

Barriers

Perceived lack of research and evidence

- "I had a conversation with my primary doctor about it shortly after the second time that I got Covid, and I just still wasn't comfortable with the kind of lack of research out there…it's just not been around long to feel like I can make an informed decision to take it."
- "I just didn't feel like it had the proper clinical trials to really push it out to the mass public."
- "lack of research on long term studies [is] my biggest thing, because I'm like 'you don't know 20 years from now, something's gonna come up'."
- "The longer that we have it out in, the more that they're able to study the effects of it long term. I think that's still kind of my hang up on it."

Self-perceived low risk

- "Covid didn't seem like something that would hurt me. I don't get the flu shot. Covid feels like the flu to me. So why would I get the Covid shot?."
- "I don't ever get the flu shot. I'm a relatively healthy person."
- "Another reason...I felt confident not getting the vaccine is the fact of how immune I had been to Covid...I already have what I need to give my baby and my breast milk."

Low perceived necessity of boosters

- "I will either be fine or I won't, and that's what it comes down to...I don't wanna this is just another thing I have to do every year...It seems it seems extra for a minimal reason...If the boosters become basically annual, I probably won't [receive them]."
- "I probably wouldn't have [gotten the booster if my doctor recommended it] because I was already getting like 60 other shots."

- "I'm just thinking how many boosters do I need of the same shot, You know. I've already gotten it, and then I've been sick with it. I'm sort of thinking, 'I think I'm okay. At this point I don't really need to keep getting the shot.'"

Perceived politicization of vaccines

- "I hate to get a vaccine based off of a political stance, and I think that to some extent that was happening."
- "[The vaccine] would have been more successful if they didn't do it in such a pushy way, and a politically charged way…They threw a Biden stamp on it, his face on it, and that affected a lot of people in my community."

Self-perceived Risk of COVID during pregnancy

Greater perceived risk

- "I had been told that…pregnant women can get more severely sick…and of course I was worried about if it would have any effects on the baby…because I don't want to put my baby at risk."
- "Early this year, when I wasn't pregnant...I was a little bit carefree like, 'Okay, bring it on. I can still try to take it'. Well, now that I'm pregnant...I don't want anything to happen to my little boy."

No perceived change in risk

- "By the time I was pregnant, I had already been around for close to 2 years, and everything was relaxing about it, and it's kind of just the…worry about getting the flu or the cold or whatever, but nothing too serious."
- "I got [COVID] while not pregnant. It just was like a cold. So I was like, okay, if I get it while pregnant, it's probably not going to do much."
- "I've had [COVID] multiple times already. I've been vaccinated and boosted, so I'm really not concerned anymore."

Sources of Information on Health Decision-making

Trusted sources

Care Providers

- "She wasn't pushy about anything. But when I had questions about it like, 'Would you recommend this? When would you recommend I get it?'...[She] gave me that information, and…encouraged me to get it."
- "I already have, of course, my own opinions, and have done some of my own research. But I look to [my doctor] for confirmation that I'm understanding what's happening."
- "I trusted my doctor because I know that they're reading all the information and the studies, and so I know that's the best place to get information."
- "I go straight to the source…to read studies, or at least the abstracts with them and try to make my own decisions on things."
- "I just mostly ask my nurses and see if they have more information available to me. If not, I'll research it up with Google Websites with '.org' is what I normally look for, instead of the '.coms'."

Family

- "With my mother-in-law being…literally the vaccine nurse, I do trust her quite a bit."
- "My mom is a researcher. And so I would a lot of times bring things to her."
- "I trust my parents' judgment a lot…so I definitely would talk about it with them."

Distrusted sources

Social Media

- "I use social media all the time otherwise, but for my COVID information I avoided it like the plague."
- "[Sources with] a low level of evidence, like Facebook or testimonial information."
- "General Facebook posts from people I don't know."

Political Sources

- “Illness should not be politically charged or involved, and so, therefore either right winged or left winged.”
- “Anything political oriented.”
- “TV news sources, big TV news places like CNN and Fox News, and all that, because they like to blow up in proportion what's actually going on around the real world.”
- “Info Wars Fox News Republicans, [and] all Republicans.”

Rural providers and news sources

- “I didn't ask [the doctor’s] office staff questions…They are vaccinated because they have to be, but I felt that it was fairly unlikely that they were going to provide very much evidence-based information.”
- “They just don't seem to keep up [with COVID].”

Vaccine Hesitancy

Vaccine hesitant

Perceived newness of the vaccine

- “I know there's lots of scientific information out there, but at the same time it's, it's something that's still very new.”
- “I’ll take the pertussis vaccine that's been being given for how many decades when you're pregnant. [But] there have literally been documented times in history when you're giving pregnant women things...that you think are safe, and then decades down the road you're like, ‘Oh, well...we shouldn't have done that. That was a bad idea’.”

Hesitancy toward other vaccines

- “We don't really get [the flu vaccine] because it seems like a lot of times it ends up being the wrong strain...you get the flu plenty often, and it never becomes too big of a deal.”
- “I’ve never really gotten vaccinated for anything since I almost died as a baby getting vaccinated. I'm more afraid of the vaccine than I am of the sickness.”

Not wanting to make decisions for children

- “I've gotten both vaccinations, but not the booster, and it's what they really wanted me to get in order for my son to get his antibodies while he was in with me. But I didn't really know how I felt about that yet. I wanted to wait until he was a bit older.”
- “It's weird when you have kids. I wasn't as worried about getting it for myself. But for [my daughter] I was like, ‘well, she's already had Covid’. Maybe we want to wait like a while longer just to have more research, and [COVID] didn't affect her badly.”

Vaccine positive

Trust in other vaccines

- “I was very convinced by what I had seen regarding its effectiveness and preventing serious illness. [The COVID vaccine] was something that I didn't really think about, any more than I thought about you know, getting my flu shot every year, or…all the routine vaccinations that I've had in my life.”
- “I don't think I've ever really thought about a vaccine as anything more than just like ‘Oh, you know, like you take medicine when you're sick. You get a vaccine when you might get sick’.”
- “These are just remarkable vaccines that, you know, are the product of a decades for the work and scientific research. I got vaccinated literally as soon as I possibly could, and...getting boosters, It's like I get a flu shot every year.”

Vaccine positivity in the broader community

- “My community is pretty open, and we're all pretty close knit together…[Our Community in Action conversations are] saying, "Hey, this is something we need to get in order to prevent it from being a widespread event again’.”

Relationship with Care Provider

Positive Relationship

Listening to Patients

- “I just go to my OB for everything, because they actually listen and understand.”
- “[they] actually listen to me and talk about it. Not just say, Well, here's what I think, and that's it.”
- “You could tell that she was listening, and she would kind of bring up sometimes stuff that we talked about in previous appointments.”
- “They're willing to...take time, and hear concerns...Ask open-ended questions. Find out what is important to the person in front of you.”

Personal relationships

- “I had the same doctor be able to deliver all 3 of my previous pregnancies...We kind of became friends outside of work, and I mean she would like, pray with me in the office, and just kind of went above and beyond as a provider.”
- “I felt like because I knew that he's my family's doctor, I...had a baseline level of trust with him already, and was able to...trust his opinion, and I didn't feel like I needed to second guess him.”
- “[My doctor] seems like someone I would want to be like friends with and I think that's important in a doctor to feel comfortable and like you trust and know them.”
- “We've got a good relationship, and she knows my health history...it doesn't feel like she's constantly looking for a computer to find what she knows about me. So...I feel comfortable asking her the bigger decisions.”

Respecting patients’ autonomy

- “It was nice to have another doctor that didn't make me feel like it was all just what they wanted me to do. Like I had some...authority, in my own care.”
- “[My doctor] was just very open to like any ideas I had, or any thoughts I had about childbirth, and wasn't super pushy about like medical interventions which I really appreciated.”
- “I didn't feel like they were...trying to persuade me either way. They were just giving me all the facts to make the best decision.”
- “They gave me...credit for like actually bringing things up that I knew what I was talking about.”
- “We've been meeting like every three weeks, and I feel like I can ask her like pretty much anything, and she's pretty honest about her opinion.”

Responsive

- “They get back to me usually within a couple of hours, if it's during the work day.”
- “I message the nurses there all the time, and they get back really quickly.”

Negative Relationship

Feeling rushed

- “I feel like I wouldn't get the best of care because I spent an hour waiting in the waiting room, but 10 minutes in the actual meeting.”
- “She could have spent more time asking for my concerns. She could have like not rushed me through my appointment. even though I waited for so long for her. Some of my appointments were actually canceled because she was taking too long with others, and I feel like that was unfair for me, because I was already in the office.”

Unresponsiveness

- “It's really difficult to..get through to my doctor's office. It usually takes a day or 2 to hear back that that's kind of a pain in the butt.”

Lack of personal relationship

- “You'll see all the doctors because anyone can deliver you...it leaves me kind of feeling like I'm floating, you know, between any one provider.”

Disregarding or belittling patient concerns

- “Post-care [my doctor] didn't like check or anything...because I had gotten stitches, because I [was] pretty open, and she didn't check them at all to see if they were gone.”
- “[The doctor] said ‘Those are normal. Those are always gonna happen. Every pregnancy is different,’ and I felt like, ‘No, there's actually something wrong’.”
